# Characterization of Movement Disorder Phenomenology in Genetically Proven, Familial Frontotemporal Lobar Degeneration: A Systematic Review and Meta-Analysis

**DOI:** 10.1371/journal.pone.0153852

**Published:** 2016-04-21

**Authors:** Carmen Gasca-Salas, Mario Masellis, Edwin Khoo, Binit B. Shah, David Fisman, Anthony E. Lang, Galit Kleiner-Fisman

**Affiliations:** 1 The Morton and ‎Gloria Shulman Movement Disorders Clinic and the Edmond J. Safra Program in Parkinson's Disease, TWH, Toronto, Canada; 2 Department of Medicine, Division of Neurology, University of Toronto, Toronto, Canada; 3 Centro integral en Neurociencias A.C. (CINAC)/HM Hospitales- Puerta del Sur, CEU-San Pablo University, Madrid, Spain; 4 Cognitive & Movement Disorders Clinic, Sunnybrook Health Sciences Centre, Toronto, Canada; 5 Dalla Lana School of Public Health, University of Toronto, Toronto, Canada; 6 Department of Neurology, University of Virginia, Charlottesville, Virginia, United States of America; 7 Jeff and Diane Ross Movement Disorders Clinic, Baycrest Center for Geriatric Health, Toronto, Canada; UCL Institute of Neurology, UNITED KINGDOM

## Abstract

**Background:**

Mutations in granulin (*PGRN*) and tau (*MAPT*), and hexanucleotide repeat expansions near the *C9orf72* genes are the most prevalent genetic causes of frontotemporal lobar degeneration. Although behavior, language and movement presentations are common, the relationship between genetic subgroup and movement disorder phenomenology is unclear.

**Objective:**

We conducted a systematic review and meta-analysis of the literature characterizing the spectrum and prevalence of movement disorders in genetic frontotemporal lobar degeneration.

**Methods:**

Electronic databases were searched using terms related to frontotemporal lobar degeneration and movement disorders. Articles were included when cases had a proven genetic cause. Study-specific prevalence estimates for clinical features were transformed using Freeman-Tukey arcsine transformation, allowing for pooled estimates of prevalence to be generated using random-effects models.

**Results:**

The mean age at onset was earlier in those with *MAPT* mutations compared to *PGRN* (p<0.001) and *C9orf72* (p = 0.024). 66.5% of subjects had an initial non-movement presentation that was most likely a behavioral syndrome (35.7%). At any point during the disease, parkinsonism was the most common movement syndrome reported in 79.8% followed by progressive supranuclear palsy (PSPS) and corticobasal (CBS) syndromes in 12.2% and 10.7%, respectively. The prevalence of movement disorder as initial presentation was higher in *MAPT* subjects (35.8%) compared to *PGRN* subjects (10.1). In those with a non-movement presentation, language disorder was more common in PGRN subjects (18.7%) compared to MAPT subjects (5.4%).

**Summary:**

This represents the first systematic review and meta-analysis of the occurrence of movement disorder phenomenology in genetic frontotemporal lobar degeneration. Standardized prospective collection of clinical information in conjunction with genetic characterization will be crucial for accurate clinico-genetic correlation.

## Introduction

Frontotemporal lobar degeneration (FTLD) is a clinically, genetically and pathologically heterogeneous group of neurodegenerative disorders. Clinical presentation is characterized by variable but progressive disturbances in behavior, cognition and language [1]. It is the fourth most common cause of dementia in people over age 65, after Alzheimer´s disease (AD), Dementia with Lewy Bodies (DLB) and vascular cognitive impairment [2], and the second most common cause of young-onset dementia after AD [3]. There is a positive family history in 30–50% of FTLD patients with at least one family member presenting with similar symptomatology. ~10–20% of FTLD cases have an autosomal dominant pattern of inheritance [1, 4, 5]. While cognitive and behavioral features have been well described, movement disorder phenomenologies have been poorly and inconsistently characterized as part of the clinical spectrum of FTLD. Despite this, the association between Parkinsonism and other movement disorder phenomenologies have been recognized since the first part of the 20th century [von Braunmuhl 1930; Akeliatis 1944]. with movement features presenting prior to, in conjunction with, or following cognitive and psychiatric symptoms [6].

Since the identification of FTLD-disease causing mutations in *MAPT* in 1998 [7], *PGRN* in 2006 [8], and hexanucleotide repeat expansions in *C9orf72* genes in 2011 [9], literature regarding clinico-genetic correlates has emerged. However, clinical descriptions are often disparate, of variable quality and detail, and in the form of single case reports or case series. Given the wide spectrum of presentations and lack of consistent reliable reporting, we examined the literature in its entirety in the form of a systematic review and meta-analysis to synthesize available data.

The objective of this work was to estimate the prevalence of clinical syndromes, and to identify trends in demographic characteristics and clinical presentations that may correlate with known genetic FTLD subgroups. Given the quest for biologic and clinical markers that could theoretically provide ante-mortem diagnosis and possibly disease modifying therapies, [10] precise clinical characterization may help identify candidates appropriate for further testing.

The results of this meta-analysis have been, in part presented in poster form at the 19th international congress of Parkinson´s disease and Movement Disorders (June 2015, San Diego, USA) and published as an abstract (http://onlinelibrary.wiley.com/doi/10.1002/mds.26295/full)

## Methods

### Selection of studies

A systematic review of the literature was performed searching PubMed and EMBASE databases and included all English language articles published from January 1, 1998 (the year of the identification of the first FTLD gene, *MAPT*) up to September 1, 2013 to identify all reports of genetically confirmed FTLD with a movement disorder spanning this time interval.

The search was restricted to the three most common FTLD pathogenic genes; *MAPT*, *PGRN* and *C9orf72*. Definition of “pathogenic” included: segregation of the gene mutation with an FTLD phenotype and/ or with pathologically-proven FTLD within a family; prediction that the mutation would be damaging to protein function consistent with the known mechanism of genetic disease; and/ or the mutation is already known to be causative of disease.

Since mutations in other FTLD-associated genes, *CHMP2B*, *VCP*, *TARDBP*, and *FUS*, are extremely rare and represent a minority of familial FTLD cases accounting for less that 1% each [11], they were excluded from analysis. Subjects with clinically typical Parkinson’s disease [12] identified to have *C9orf72* expansions that were deemed of unclear significance or incidental and not clearly causal, were excluded from analysis[13–15]. We did not stratify based on specific mutation genotype in *PRGN* or length of *C9orf72* hexanucleotide repeats for the following reasons: 1) *PRGN* mutations have a uniform pathogenic mechanism of haploinsufficiency [8], and 2) there appears to be no association between hexanucleotide repeat expansion length in *C9orf72* and clinical syndrome [12]. Since it is known that the majority of pathogenic mutations in *MAPT* occur in exons 9 through 13, we examined only cases with these exonic mutations; power issues prevented us from analyzing individual *MAPT* mutations [4]. In addition, insufficient power prevented us from analyzing in a meaningful way the prevalence of the movement disorder phenomenology observed in any of the specific genotype sub-groups for each of the three genes.

In summary, we examined the occurrence of specific movement disorder phenomenology in individuals or patient series that confirmed any mutation in *PGRN* or *MAPT* determined to be pathogenic, or in those with *C9orf72* repeat expansions greater than 30.

The search engines were queried using the terms illustrated in **Table 1** including a combination of every “A” + every “B” term. Titles and abstracts that described movement disorder features in the context of genetically proven FTLD were flagged and the full articles were reviewed.

**Table 1:** Search terms used for PubMed and EMBASE searches.

A	B
Frontotemporal lobar degeneration	Parkinsonism
Frontotemporal dementia	Dystonia
Motor neuron disease	Stereotypy/stereotypical movements
Semantic dementia	Tic
Progressive nonfluent aphasia	Myoclonus
Progranulin	Gait
PGRN	Corticobasal syndrome/disease
GRN	Tremor
(FTDP-17/FTDP17)	Progressive supranuclear palsy
(FTD-U)	Chorea
(MAPT)	Movement disorder
(C9orf72)	

Inclusion criteria included: (i) movement disorder at some point in disease course; (ii) detailed clinical description of movement disorder phenomenology; (iii) FTLD proven genetically (*MAPT*, *PRGN*, *C9orf72* causative variants); (iv) human subjects; (v) papers published in English; and (vi) inclusion and presentation of data sufficient for estimation of the proportion of patients presenting with outcome of interest. Exclusion criteria included: (i) lack of movement disorder as part of clinical presentation; (ii) lack of description of movement disorder phenomenology; (iii) clinical information not presented individually or in ratios (i.e. data must have been presented in a way that showed frequency of clinical features); (iv) absence of genetic confirmation of FTLD; (v) animal or in vitro data without human subjects; (vi) previously reported data.

Abstracts were verified by two independent reviewers (BBS, CG). In cases where insufficient information was provided to determine eligibility for inclusion, the full article was reviewed. Some subjects were reported more than once in different publications and when uncertainty existed, the authors were contacted to ensure duplication of reporting did not occur. A manual search of references from included publications was performed. Those studies that met inclusion criteria and that were not already identified through the database query were included in the meta-analysis.

### Data extraction

Data extraction was performed in duplicate using a standard assessment form by two investigators (BBS, CG). Any differences among results were discussed among co-authors (GKF, MM, AEL) until consensus was achieved. In addition to genetic data, demographic and disease specific clinical characteristics of movement disorder and other features were collected. These included:

1. Average age of symptom onset, gender, and duration of symptoms
2. Initial presentation (movement, non-movement or both)
  - Non-movement syndromes included behavioral, language or other cognitive disorder or any combination of these
3. Prevalence of MD “syndromes”
  - Parkinsonism defined as bradykinesia and rigidity
  - Progressive supranuclear palsy syndrome (PSPS) syndrome defined as vertical gaze abnormality, and either axial rigidity, OR postural instability.
  - Corticobasal syndrome (CBS) defined as asymmetry (any one of dystonia, rigidity, bradykinesia) AND at least one cortical feature including myoclonus, cortical sensory loss, limb apraxia or aphasia.
4. Levodopa-response classified as absent, partial or good.

We attempted to extract information in isolation of specific MD syndromes, such as presence of dystonia, myoclonus, etc., but this was not possible since the calculated probabilities are derived from study-level data and there is no way to stratify these probabilities in isolation for an individual phenomenology. For example, if a study had 10 subjects and the probability of Parkinsonism was 0.4 and the probability of rigidity was 0.3 and the probability of bradykinesia was 0.2, there was no way to tell which of the 10 subjects only had one of the phenomenologies.

Similarly, we chose to identify the most common and recognized clinical PSPS syndrome (“Richardson syndrome”) and were not able to stratify the other variant subtypes of PSPS [PSP-CBS, PSP with pure akinesia with gait freezing (PAGF)] for similar reasons.

### Quality of the literature

A quality assessment was performed for each study based on criteria developed by the investigators. Studies were assigned one point for each question answered “Yes”:

1. ≥ 5 subjects
2. Details of movement disorder phenomenology in the first 3 years of the disease in at least 50% of the sample. If the movement disorder phenomenology appeared after three years or if a study did not specify when the movement disorder occurred in the course of the illness, no point was assigned.
3. Longitudinal follow-up of ≥ 5 years

### Statistical analysis

For descriptive statistics, one-way ANOVA was used to assess for statistical differences between genetic subgroups for continuous variables. Fisher’s exact test was used for categorical variables. A p-value of ≤0.05 was considered statistically significant.

### Case studies

To allow for inclusion of the large number of case studies (reports based on single patients) populating the current literature, case studies were combined to form a single patient population, which was treated as a publication for meta-analytic purposes. For each outcome of interest, the number of patients with and without the outcome within the artificial study was used to obtain the study-specific prevalence for the outcome.

### Meta-analysis

Summary pooled estimates of the extracted prevalence data were obtained by conducting random-effects meta-analyses using the DerSimonian and Laird method [16]. Prevalence data for initial presentation (movement, non-movement or both), syndromes, and individual phenomenologies from individual studies were pooled together by first using the Freeman-Tukey arcsine transformation [17] The variance was calculated as $\frac{1}{\left(N_{total}+1\right)}$, where *N*_*total*_ was the number of patients in the study, and the transformed prevalence estimates were converted back as ${\left(sin\;\left(\frac{N_{transformed}}{2}\right)\right)}^{2}$, where *N*_*transformed*_ was the transformed estimate [18]. Summary pooled estimates were presented as percentages. As a secondary outcome, the analysis was also stratified by *MAPT*, *PGRN*, and *C9orf72* genetic mutations to examine for potential trends.

Between-study heterogeneity was assessed using the and I^2^ statistic. Confidence intervals (CIs) for I^2^ statistic were also calculated to quantify the uncertainty in the heterogeneity estimates [19] Substantial heterogeneity was considered to be present if the I^2^ statistic or confidence intervals were ≥ 50% (http://handbook.cochrane.org/chapter_9/9_5_2_identifying_and_measuring_heterogeneity.htm). Additionally, the H statistic and confidence intervals were also calculated to support the heterogeneity assessment, with a value of 1 indicating homogeneity [20]

In addition to quantifying the heterogeneity, summary pooled estimates for initial presentation, syndromes, and phenomenologies were recalculated under the assumption of substantial heterogeneity (I^2^ = 90%) in order to assess the impact of undetected heterogeneity on estimates [21].

Publication bias was a concern due to the inclusion of case studies, which may represent the most extreme presentations of FTLD. Each outcome of interest was assessed graphically using funnel plots where the logit of the outcome was plotted against the study variance. Egger’s test for funnel plot asymmetry was used to statistically assess for publication bias only where there were at least 10 studies [22]. A p-value of ≤0.05 was considered statistically significant.

We performed sensitivity analyses evaluating the impact of including the pooled case studies by repeating the main analysis without them. Values from the main analysis and the sensitivity analysis were compared to assess the influence of the inclusion/exclusion of the case studies.

All statistical analyses were conducted in Stata version 12.1 (Stata Corp., College Station, TX).

## Results

**Fig 1:**
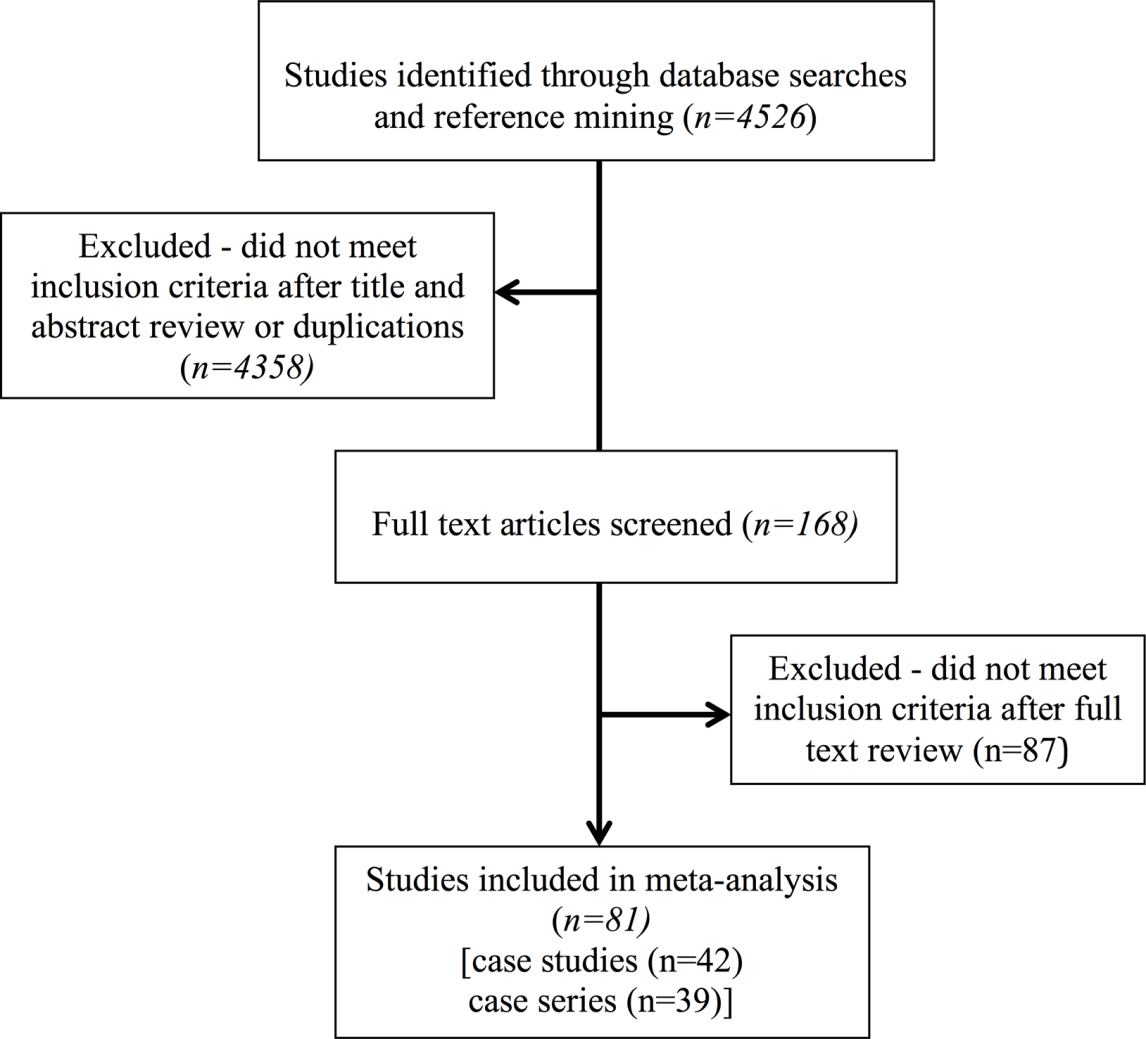
Flow diagram of the study selection process.

The combined MEDLINE and EMBASE searches yielded 4526 original titles **(Fig 1,** Prisma Flow Diagram). 168 full text-articles were reviewed, including those that did not include an abstract and could not be excluded form reviewing the title; 87 articles were excluded due to insufficient clinical data **(S1 Table)**. A total of 77 distinct studies were included in the meta-analysis [23–99].

However, for the purpose of data collection, in those studies that included mutations in more than one gene (4 studies), each mutation and clinical information related to that mutation, was treated as a separate study. Using this method, a total of 81 studies were included in this systematic review. Of these 42 were case series (n ≥ 2) and 39 were individual case studies (n = 1). There were a total of 376 patients included from all studies. The proportion of cases with mutations in *MAPT*, *PGRN*, and *C9orf72* expansions was 44.2%, 31.7%, and 24.2%, respectively.

### Demographic Characteristics

**Table 2** outlines the demographic characteristics of study subjects. The mean age at onset for all subjects with genetically confirmed FTLD and a movement disorder was 51.7 years old. Men and women were represented approximately equally with an average disease duration spanning the time from symptom onset till death of 7.1 years.

**Table 2:** Demographic characteristics of study subjects stratified based on genetic subgroup.

	MAPT	PGRN	C9ORF72	Overall
**No. of patients: *Frequency* (%)** ^A^	166 (44.1)	119 (31.7)	91 (24.2)	376 (100.0)
**No. of patients per study: *mean* (min, max)** ^B^	7.2 (2.0, 25.0)	10.0 (2.0, 34.0)	8.3 (2.0, 40.0)	8.6 (2.0, 40.0)
**Age at onset: *Mean years* (min, max)** ^C^	45.8 (28.0, 63.5)	59.6 (54.8, 68.5)	54.7 (42.3, 70.5)	51.7 (28.0, 70.5)
**Disease duration: *Mean years* (min, max)** ^D^	6.5 (0.7, 16.0)	6.9 (4.9, 10.0)	8.0 (2.1, 16.2)	7.1 (0.7, 16.2)
**Proportion of males: % (95% CI)** ^E^	50.7 (38.0–63.4)	42.7 (31.8–54.0)	41.7 (25.1–59.3)	45.7 (37.9–53.7)

^A^ Fischer’s exact test: p = 0.315, therefore not statistically significant.

^B^ Fischer’s exact test: p = 0.196, therefore not statistically significant.

^C^ One-way ANOVA: p<0.001; MAPT vs. PGRN: <0.001; MAPT vs. C9ORF72: 0.024; PGRN vs. C9ORF72: 0.126.

^D^ One-way ANOVA: p = 0.860, therefore not statistically significant.

^E^ Estimates from random-effects meta-analyses.

There was a significant difference between the age at onset stratified by genetic mutation (p<0.001). The age at onset was significantly earlier in patients with *MAPT* compared to *PGRN* (p<0.001) and *C9orf72* (p = 0.024). The age at onset was not statistically different between *PGRN* and *C9orf72* (p = 0.126). No other demographic characteristics differed significantly by genetic mutation.

### Initial presentation

Characteristics of subjects upon initial presentation are outlined in **Table 3**. 27.1% (95% CI 17.4–37.9%) of subjects were identified to have their first manifestation of illness as a movement disorder (preceding cognitive or behavioral symptoms). Except for 4 subjects with *C9orf72* mutations presenting with motor neuron disease (MND) [37, 85] and another 2 subjects also with *C9orf72* mutations presenting with MD and MND [82, 85], all subjects with movement symptoms at onset presented with a movement disorder including clinical syndromes of Parkinsonism, CBS or PSPS. We will not comment further or present data on patients with a pure MND presentation.

**Table 3:** Initial Presentation stratified based on genetic subgroup.

	MAPT % (95% CI)	PGRN % (95% CI)	C9ORF72% (95% CI)	Overall % (95% CI)
**Movement Disorder**	35.8 (18.9–54.8)	10.1 (4.8–17.1)	34.0 (14.9–56.3)	27.1 (17.4–37.9)
**Non-movement Disorder**	62.7 (44.0–79.6)	83.6 (73.8–91.5)	46.2 (17.5–76.3)	66.5 (54.0–78.0)
**Movement + Non-movement Disorder**	5.8 (2.4–10.4)	7.2 (2.9–13.4)	15.1 (5.7–28.1)	7.7 (4.8–11.1)

Note: 13 studies missing data on initial presentation necessary to calculate percentage with each type of initial presentation. 3 studies with incomplete data on initial presentation.

Due to the random effects meta-analysis, the studies are being given different weights dependent on the sample size so the overall number of subjects do not sum to 100%. The studies with missing data are very small so they do not add much weight to the estimates.

The number of studies used to calculate the summary pooled estimate for each outcome of interest can be seen in **S2 Table.** In ~66.5% (95% CI 54.0–78.0%) of subjects, the initial presentation was categorized as non-movement. Subjects whose initial presentation was non-movement most commonly manifested a behavioral syndrome (35.7%, 95% CI 24.4–47.9%), while cognitive (14.4%, 95% CI 8.7–21.2%) and language (9.9%, 95% CI 6.6–13.8%) presentations were less frequent. Some of the non-movement presentations were variable combinations of behavioral, cognitive and language symptoms (**S3 Table**).

Pooled estimates for movement disorder presentation differed between *MAPT* and *PGRN* mutations. 35.8% (95% CI 18.9–54.8%) of subjects with *MAPT* presented with an initial movement disorder, whereas 10.1% (95% CI 4.8–17.1%) of subjects with *PGRN* had an initial movement disorder. Additionally, 5.4% (95% CI 2.2–10.0%) of subjects with genetic mutation *MAPT* presented with a language disorder, in contrast to 18.7% (95% CI 11.5%-27.3%) of subjects with *PGRN*. For both of these outcomes, the 95% CIs for the estimates stratified by genetic mutation did not overlap, indicating a statistically significant difference (which cannot be ruled out when CIs overlap) [100].

### Parkinsonism, Corticobasal (CBS) and Progressive Supranuclear Palsy Syndromes (PSPS)

Parkinsonism was the most common movement disorder syndrome reported in 79.8% of subjects (95% CI 69.7–88.2%) followed by PSPS (12.2%, 95% CI 6.2–19.7%) and CBS (10.7%, 95% CI 6.7–15.4%), respectively, at any given time during the course of the disease (**Table 4**).

**Table 4:** Movement disorder syndromes present at any point during the FTLD disease course stratified based on genetic subgroup.

	MAPT % (95% CI)	PGRN % (95% CI)	C9ORF72% (95% CI)	Overall % (95% CI)
**PSPS**	17.4 (5.8–33.5)	8.1 (1.8–18.3)	6.0 (2.1–11.9)	12.2 (6.2–19.7)
**CBS**	7.6 (3.7–12.8)	26.4 (10.6–46.3)	6.1 (2.3–11.6)	10.7 (6.7–15.4)
**Parkinsonism**	79.9 (63.8–92.1)	71.3 (54.7–85.4)	91.4 (81.3–97.8)	79.8 (69.7–88.2)

### Levodopa Responsiveness

Response to levodopa is summarized in **S1 Fig** comprising a total of 63 distinct subjects in 25 studies. Overall levodopa response was good in 15.3% (95% CI 4.2–31.6%) of patients reported, partial in 21.9% (95% CI 7.7–40.8%) and absent in 50.9% (95%CI 23.3–78.3%) **(S4 Table)**.

### Literature Quality

25.9% of the studies had 5 or more subjects **(S5 Table)**; 69.1% of studies had detailed information regarding the movement disorder phenomenology in the first 3 years; 44.4% of studies reported clinical information for 5 or more years.

### Heterogeneity and Publication Bias

The heterogeneity was quantified for overall outcomes and is presented in **S6 Table**. Based on an established threshold of ≥ 50% for the I^2^ statistic, most estimates were found to have substantial heterogeneity. For movement disorder at initial presentation, the proportion of variation due to heterogeneity between studies was 73.8% (95% CI 63.7–81.1%). Initial presentation as non-movement disorder had a similar result with the variation resulting from between-study heterogeneity being 75.4% (95% CI 65.8–82.3%). For the subset with non-movement disorder at initial presentation that manifested as a behavioral syndrome, the between study variation from heterogeneity was 73.2% (95%CI 62.4–80.9).

Parkinsonism and PSPS also both had I^2^ statistics that were greater than 50% indicating substantial heterogeneity. Estimates for levodopa response that were absent or partial were 78.2% (95% CI 57.0–88.9%) and 58.3% (95% CI 8.8–81.0%) respectively. With the exception of partial levodopa response, the I^2^ 95% CIs were above 50% for the outcomes mentioned, indicating with high certainty that substantial heterogeneity was present for these outcomes. Performing the H statistic replicated these findings, with the H statistic and 95% CIs above 1.5.

The impact of the heterogeneity was assessed by repeating the main analysis under the assumption that the proportion of between study variation due to heterogeneity was 90%. The results are presented in **S7 Table**. The largest differences were found for levodopa response, CBS, and cognitive and language presentations. However, all of the differences were less than 2%, with the largest for levodopa response (present) being 1.9%.

Finally, meta-analyses of the main outcomes were conducted without the inclusion of pooled case studies. Pooled summary estimates along with the quantified heterogeneity are presented in **S8 Table**. When comparing the results from the main analysis with the sensitivity analysis, the 95% CIs for all the estimates overlapped. Similar to the analysis comparing genetic mutations, statistical significance cannot be ruled out when CIs overlap. However, none of the estimates were necessarily statistically different.

There was evidence of publication bias for two outcomes of interest where there were more than 10 studies included: behavioral + cognitive disorder at presentation and Parkinsonism with a larger number of smaller studies having a lower proportion of behavioural + cognitive disorder reported (p<0.001) **(S2 Fig)**. With respect to Parkinsonism, there was a larger number of smaller studies having a higher proportion of Parkinsonism reported (p<0.001) **(S3 Fig**).

## Discussion

The focus of this systematic review and meta-analysis was to determine prevalence of movement disorder phenomenology in people with genetically proven FTLD and manifesting a movement disorder during the disease course, and also to explore whether genetic mutation predicted clinical characteristics. It was not within the scope of this review to determine prevalence of movement disorders in all cases of genetically proven FTLD as those without movement disorders were not included in the cohort. We also chose to exclude studies that had only pathologically proven FTLD without genetic confirmation to manage the scope of our study, though pathological data was collected and will be the subject of a separate manuscript.

While the published literature is variable in quality and comprised mostly of retrospective case reports and small case series, this first comprehensive review synthesizes and summarizes trends in movement disorders occurring in genetically confirmed FTLD in the available literature.

### Prevalence of genetic mutations

*PGRN*, *MAPT* and *C9orf72* gene variants account for at least 17% of total FTLD cases [101], and between 32–40% of all identified genetic causes of FTLD [9]. *PGRN and MAPT* are estimated to account for 5–20% of familial FTLD cases, and *C9orf72* mutations account for ~21% of familial FTLD cases [102] The genetic mutations thought to contribute to the remainder of familial FTLDs (~60%) confirmed by positive family history are rare or as yet undiscovered [103].

*PGRN* is found causative in an additional 1–5% of sporadic FTLD cases. C9*orf*72 is also responsible for related syndromes occurring in 6% of sporadic FTLD cases, 37% of familial ALS cases, and 6% of sporadic ALS cases [102, 104]. In all series, the *C9orf72* repeat expansions have been the most common genetic cause of familial ALS (more frequent than *SOD1* mutations).

In our highly selected population (cases of *MAPT*, *PGRN* and *C9orf72* causing FTLD and a movement disorder), the most common gene involved was *MAPT* with a prevalence of 44% followed by *PGRN* (32%) and *C9orf72* (24%). Since *MAPT* mutations were first identified in 1998, 8 years before *PGRN* was discovered, and 13 years before *C9orf72* was discovered, the large number of papers dealing with *MAPT* mutations may have artificially skewed the prevalence findings to appear that MAPT has a significantly higher proportion of movement disorders in its clinical presentation.

### Age at onset

Age at onset of familial FTLD has been reported to differ depending on genetic mutation with *PGRN* presenting on average at age 59 [105] and *C9orf72* at age 56 [52] whereas *MAPT* presents on average at age 49 [106]. Similarly, in our subset of patients with FTLD and a movement disorder, *MAPT* patients presented at age 46, *PGRN* at age 60, and *C9orf72* at age 55 (**Table 2)**. Significant differences were found between *MAPT* and *PGRN* and between *MAPT* and *C9orf72*. There was no significant difference between *PGRN* and *C9orf72*.

### Disease duration

Disease duration in FTLD has been reported to be approximately 7 years in *PGRN* and *MAPT* [106] and 5 years in *C9orf72*. This can be explained by the high frequency of MND/ALS reported among FTLD-*C9orf72* carriers (> 40%) [52]. In contrast, our cohort showed the opposite trend with *C9orf72* mutation carriers having an average disease duration of 8 years; though there were no statistically significant differences between genetic subgroup, the trend towards a longer disease duration in the *C9orf72* mutation carriers would seem counter intuitive and contrasts with other reports. One possible explanation is the fact that in this population of genetically-confirmed FTLD patients with a MD, there was a low frequency of MND in the *C9orf72* population (6/91~7% of the cohort). Still the lack of statistical difference between *C9orf72* subjects and *MAPT/PGRN* is likely artefactual and may represent a power issue. The number of *C9orf72* subjects was lower (though the difference was not statistically significant) than the subjects manifesting the other mutations and so may be too small to accurately reflect a real difference.

### Initial presentation

FTLD has been reported to present as a primary language deficit [progressive non-fluent aphasia (PNFA), semantic dementia (SD)] or a behavioral variant (bvFTD); bvFTD has the highest prevalence representing 50–70% of all the FTLD cases [1,2,4]. The majority of subjects in our cohort also reported behavioral, cognitive or language abnormalities as a defining initial feature of illness (67%); an additional 8% of studies had combined non-movement and movement symptoms at initial presentation. 27% of subjects presented with a MD as the first manifestation of illness.

Movement disorder as initial presentation was higher in *MAPT* subjects (36%) compared to subjects with *PGRN* mutation (10%). In studies with non-movement manifestations as initial presentation, language disorder was less common in *MAPT* subjects (5%) compared to subjects with *PGRN* mutation (19%). Other differences between genetic mutation could not be determined due to the 95% CIs overlapping between summary pooled outcomes.

### Movement Disorders

#### Parkinsonism

The most common movement disorder syndrome associated with all FTLD is Parkinsonism. The prevalence of Parkinsonism in patients with FTLD reported in the literature varies widely between 6%-30% [6, 107, 108]. The phenomenology includes axial and limb rigidity, bradykinesia and postural instability. Resting tremor is usually absent [11], although other types of tremor are not unusual [52, 109]. Levodopa responsiveness is rare but does occur, generally with an initial good, but transient, or only partial response [11, 110]. Comparing these previous reports to our population is difficult given that all studies included in the meta-analysis, by definition had a movement disorder reported at some point in the disease course. In this context, Parkinsonism was also the most common syndrome reported in ~80% of studies; the prevalence of Parkinsonism appeared uniform across genetic mutations with no one gene being necessarily associated with a higher prevalence of Parkinsonism than the others due to the overlapping 95% CIs of the summary pooled estimates. In our cohort, ~37% of the patients receiving L-dopa had at least a partial response though this may be strongly influenced by reporting bias as many of the studies did not mention whether a trial of L-dopa was undertaken.

#### CBS and PSP-like syndromes

The prevalence of PSPS and CBS in the context of FTLD has not been well studied. One retrospective clinical study looking at the distribution of clinical syndromes in FTLD found that CBS and PSPS combined were present in 8.6% of the cohort representing only a small proportion of all FTLD syndromes [111]. PSPS is typically associated with tau pathology while CBS is recognized to be associated with variable underlying histopathology including tau, TDP-43 and Alzheimer’s disease [112]. With respect to genetically defined FTLD, CBS has been most often associated with *PGRN* mutations though the prevalence of CBS due to *PGRN* is not known. In our meta-analysis, CBS was identified in ~11% of patients of genetic FTLD combined with MD. CBS appeared to occur more commonly with *PGRN* mutations compared to *MAPT* and *C9orf72* mutations, however the 95% CIs overlapped so conclusions are limited.

PSPS in our cohort was reported in ~12% of patients. We were unable to confirm differences in presentation of PSPS by genetic mutation due to the 95% CIs overlapping for summary pooled estimates between genetic subgroups. In previous literature, PSPS has been most often associated with *MAPT* mutations [51, 81, 113] and rarely reported due to *PGRN* mutations [63, 77, 88] or *C9orf72* expansions [59, 78].

### Heterogeneity

Evidence of heterogeneity was found for some pooled estimates from the main analysis. Movement disorders, non-movement disorders, behavioral disorders, PSPS, Parkinsonism, and levodopa responsiveness (that was absent) all had I^2^ statistics above 50%. Additionally, the 95% CIs for the statistic were also above 50% indicating that there was substantial variation in estimates between studies due to heterogeneity. Similarly, the H statistic, which is generally stable and independent of the number of studies included in the analysis, confirmed the heterogeneity for these outcomes with the 95% CIs being ≥ 1.5 [20].

When meta-analyses were conducted under the assumption of substantial heterogeneity (I^2^ = 90%), estimates were consistent with the main analysis. This indicates that any undetected heterogeneity in the main analysis seems to have a minimal impact on estimates. The biggest absolute percent difference was 1.9% for levodopa responsiveness that was present. As expected, for the outcomes where high heterogeneity was detected in the main analysis, the percent difference was minimal when the I^2^ was assumed to be 90%.

The impact of including the case studies (reports on single patients) was also assessed by excluding them and re-running the analyses. The estimates appeared to be similar without the case studies. Importantly, the same outcomes were found to have substantial heterogeneity as when the case studies were included. This indicates that the inclusion or exclusion of them alone is unlikely to explain the detected heterogeneity.

Further work is needed to explore and understand the detected heterogeneity in the outcomes examined in this paper. Genetic subgroup may be a potential factor, however this could not be firmly concluded in this analysis due to the small number of studies available stratifying by genetic mutation. While some outcomes such as movement disorder and language disorder at initial presentation were found to be statistically different between subjects with *MAPT* and *PGRN* mutations due to the non-overlapping 95% CIs, it is uncertain whether there are other statistical differences for the other outcomes by genetic mutation. More work is required to explore these outcomes by genetic subgroup as well as other possible factors that may explain the heterogeneity. The detected heterogeneity could reflect truly heterogeneous clinical features of genetic FTLD, variability in methods and measurement by investigators across studies, or heterogeneity that is attributable to some other study attribute that was not identified or recorded. It is important to interpret pooled estimates from multiple studies with caution where heterogeneity is present, as average effects across studies may provide a poor representation of the effects in individual subpopulations.

### Quality of the literature

One variable we examined when assessing quality of the literature was description of movement disorder phenomenology within the first three years of the illness. It is well recognized that as neurodegenerative diseases advance, regardless of the underlying pathogenesis or causative genetic mutation there is a common later-stage syndrome of rigidity, immobility and eventually progression to the bed bound state [114] As such, we were primarily interested in how the illness presented in order to theoretically help direct evaluations for the purpose of diagnosis. Once a full blown cognitive syndrome manifested, a diagnosis was likely already determined.

Other factors we used to determine whether the literature was of good quality related to number of cases in a study; single case reports tend to report greater detail but often are unusual presentations and not necessarily representative of a population. Given the fact that advances in this field are relatively recent, prospective, large population studies outlining natural history, clinical features, genetic and pathologic studies are uncommon and therefore our results must be interpreted with caution. While this is the first systematic review and meta-analysis to synthesize available data regarding genetic subgroups and movement disorders in genetic FTLD, the conclusions are based on imperfect data.

### Other limitations

We included standard criteria for definitions of the three syndromes of Parkinsonism, PSPS and CBS. While we were interested in analyzing the frequency of individual features such as tremor, dystonia and other movement disorder phenomenology when not part of a clinical syndrome (PSP/CBS/Parkinsonism), it was not possible to isolate the individual features from the clinical syndromes based on the data available from the literature. As such, documenting prevalence of phenomenology exclusive of reported syndromes was not possible.

Finally, statistical adjustments for multiple tests were not used for this study; the Bonferroni adjustment was not used to assess the potential difference in onset age between different genetic mutations due to the fact that each outcome was assessed individually (not universally in combination with all others), and to avoid Type II error [115, 116].

This study is the first to systematically describe the clinical presentation of the commonest genetic forms of FTLD in the context of movement disorders though variability and heterogeniety of available literature prevents definitive conclusions.

### Publication bias

Statistical testing and graphical exploration of the data found evidence of publication bias for behavioral + cognitive disorder at presentation and Parkinsonism. Due to this, pooled estimates for these outcomes should be interpreted with caution as the data gathered for these outcomes may not be accurate. The explanation for this may be due to the selection criteria applied for this review. We identified and reviewed more papers relating to our systematic search on *MAPT* mutations followed closely by those on *PGRN* mutations and then less frequently *C9orf72* hexanucleotide expansions. This likely represents a publication bias relating to time of discovery and publication of the specific mutations. Additionally, this review was restricted to studies published in English, which may have systematically excluded studies from this review.

## Conclusions

In conclusion, this is the first systematic review and meta-analysis of the occurrence of movement disorder phenomenology in genetic FTLD. We found that Parkinsonism was the most common, whereas CBS and PSPS were much less frequent. Subjects with MAPT more commonly presented with a movement disorder compared to those with PGRN mutation.

Standardized prospective collection of clinical information in conjunction with genetic characterization will be crucial for more accurate clinic-genetic correlation in future studies.

## Supporting Information

S1 Fig Number of studies and patients where L-dopa responsiveness was assessed. Flow chart describes the number of studies that did and did not assess L-dopa responsiveness along with the number of patients that was assessed. Complete information means enough data was available to determine the percentage of patients whose response was absent, partial, and good. Missing information means that only some information on patients was available or no information on L-dopa responsiveness was available.
(PDF)

S2 Fig Publication bias for behavioural + cognitive onset. (PDF)

S3 Fig Publication bias for parkinsonism. (PDF)

S1 Table Eighty-seven full-text excluded articles and the reasons for exclusión. (DOCX)

S2 Table Number of studies for each outcome. (DOCX)

S3 Table Non-motor presentations. (DOCX)

S4 Table Levodopa responsiveness. (DOCX)

S5 Table Quality of studies. (DOCX)

S6 Table Quantifying heterogeneity of pooled estimates. (DOCX)

S7 Table Examining impact of substantial heterogeneity on pooled estimates. (DOCX)

S8 Table Examining impact of removing pooled case studies. (DOCX)
