# Supplementary material for: Characterization of Movement Disorder Phenomenology in Genetically Proven, Familial Frontotemporal Lobar Degeneration: A Systematic Review and Meta-Analysis
Source: PLoS One. 2016 Apr 21;11(4):e0153852. doi: 10.1371/journal.pone.0153852 (PMC4839564; doi:10.1371/journal.pone.0153852)
Supplement: S1 Fig — Flow chart describes the number of studies that did and did not assess L-dopa responsiveness along with the number of patients that was assessed. Complete information means enough data was available to determine the percentage of patients whose response was absent, partial, and good. Missing information means that only some information on patients was available or no information on L-dopa responsiveness was available. (PDF) [file pone.0153852.s001.pdf]

Supplementary figure 1. Number of studies and patients where L-dopa responsiveness was assessed

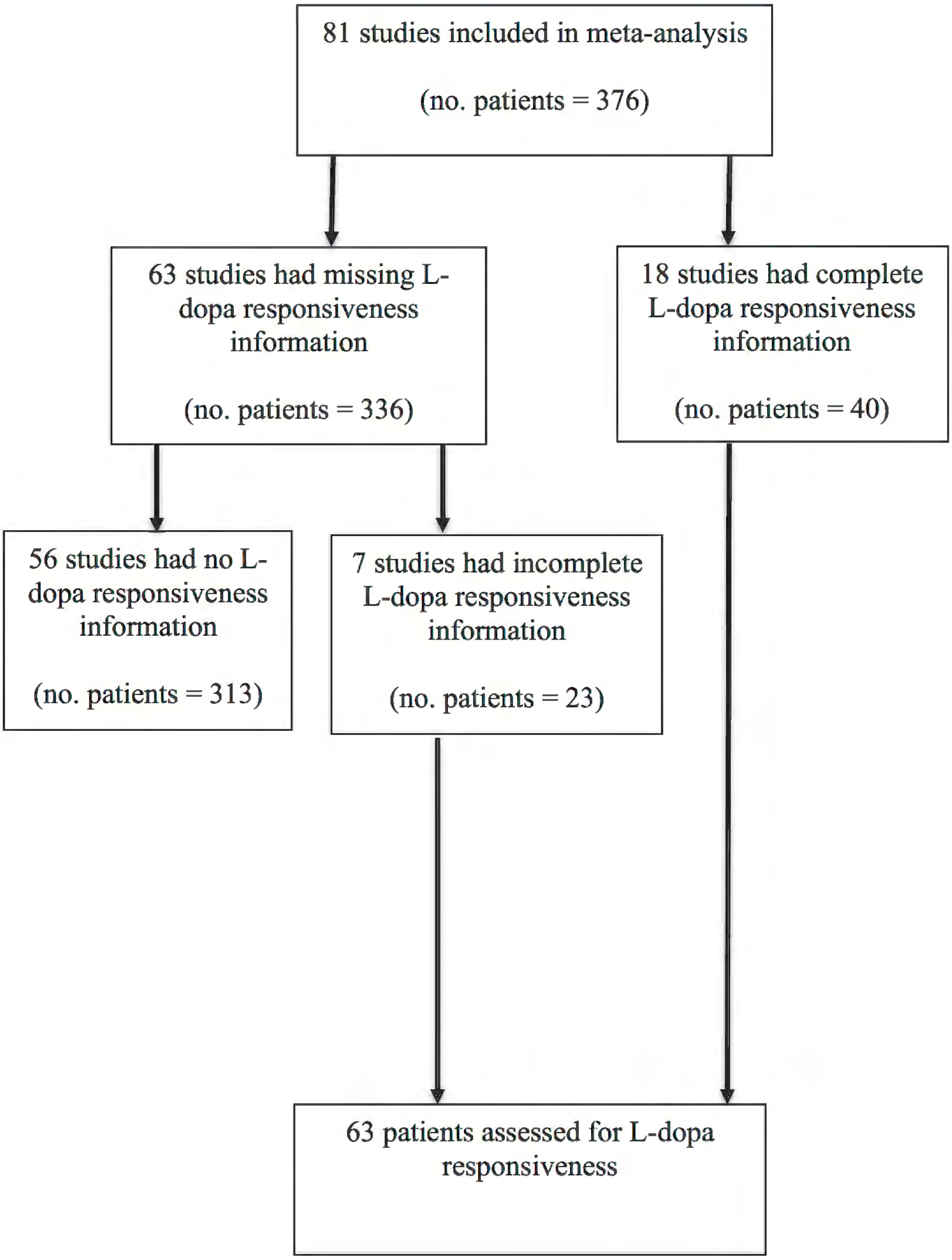

Flow chart describes the number of studies that did and did not assess L-dopa responsiveness along with the number of patients that was assessed. Complete information means enough data was available to determine the percentage of patients whose response was absent, partial, and good. Missing information means that only some information on patients was available or no information on L-dopa responsiveness was available.
