# Supplementary figures and images for: Characterization of Movement Disorder Phenomenology in Genetically Proven, Familial Frontotemporal Lobar Degeneration: A Systematic Review and Meta-Analysis
Source: PLoS One. 2016 Apr 21;11(4):e0153852. doi: 10.1371/journal.pone.0153852 (PMC4839564; doi:10.1371/journal.pone.0153852)

Supplementary figure 2. Publication bias for behavioural + cognitive onset

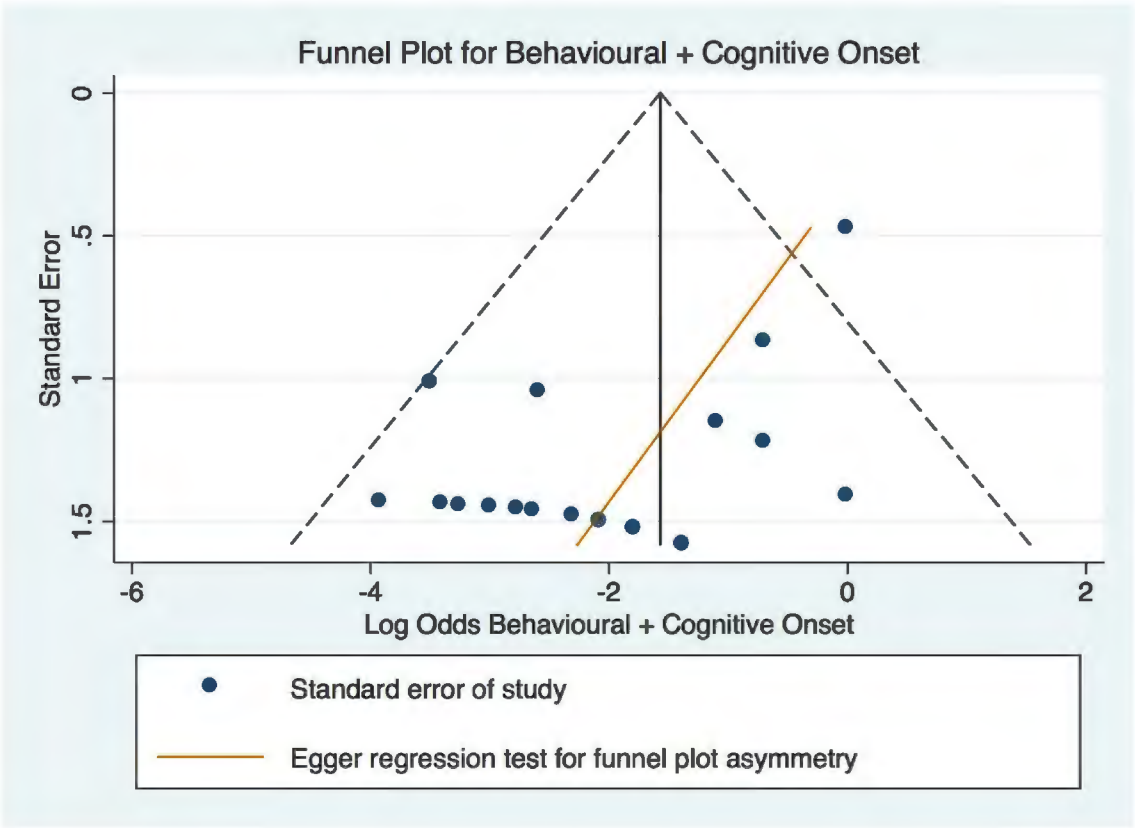

Supplement: S2 Fig — (PDF) [file pone.0153852.s002.pdf]

**Supplementary figure 3. Publication bias for parkinsonism**

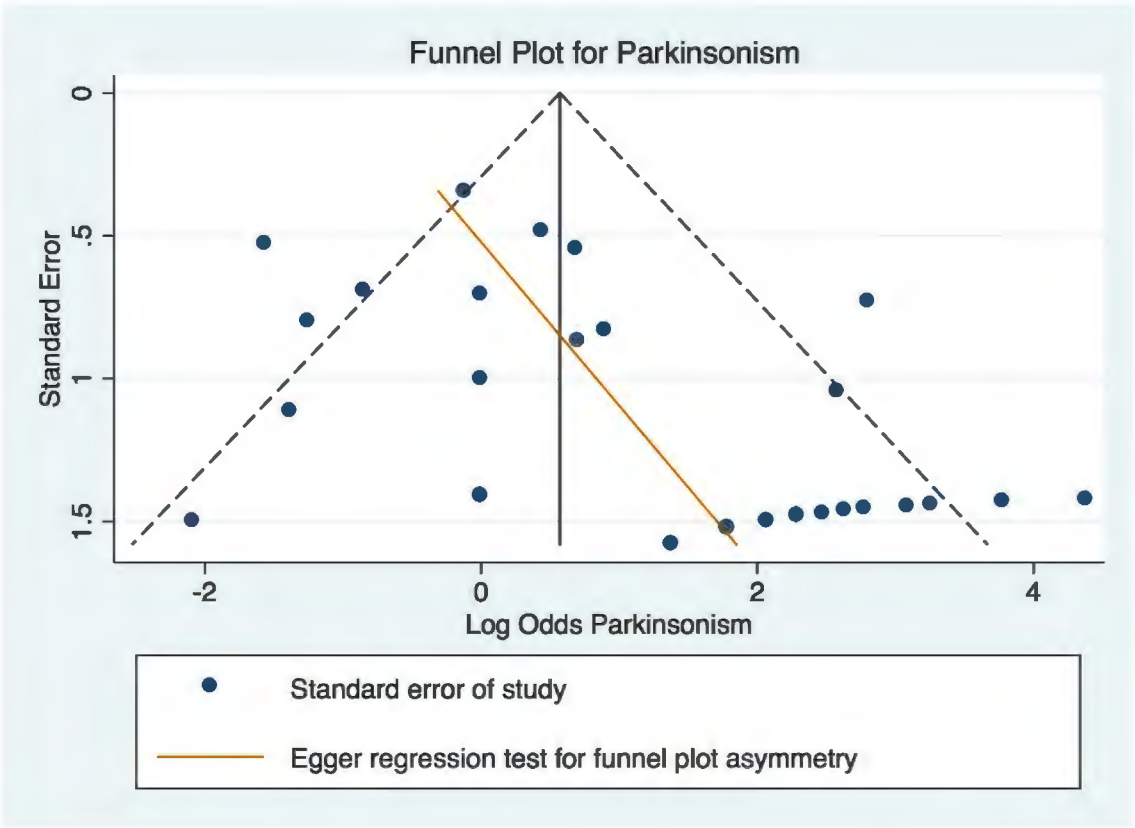

Supplement: S3 Fig — (PDF) [file pone.0153852.s003.pdf]
