# Supplementary material for: Characterization of Movement Disorder Phenomenology in Genetically Proven, Familial Frontotemporal Lobar Degeneration: A Systematic Review and Meta-Analysis
Source: PLoS One. 2016 Apr 21;11(4):e0153852. doi: 10.1371/journal.pone.0153852 (PMC4839564; doi:10.1371/journal.pone.0153852)
Supplement: S1 Table — (DOCX) [file pone.0153852.s004.docx]

**S1. Table.** Eighty-seven full-text excluded articles and the reasons for exclusion.

| **Reason for exclusion** | **Articles excluded** |
| --- | --- |
| Inadequate clinical data | 1,2,3,4, 9, 14, 20, 21,26, 27, 29, 31, 37, 39, 40, 42, 46, 47, 53, 54, 61 |
| No movement disorder description | 5, 17, 22, 23, 38, 59, 60, 66, 72, 78 |
| No distinct diagnosis | 6 |
| Did not meet genetic inclusion criteria. | 7, 11, 13, 15, 55, 67, 69, 70, 71, 73, 74, 75, 79, 83 |
| No FTLD subjects | 8, 12, 16,19, 24, 25, 28, 30, 33, 34, 36, 48 |
| Linkage-analysis only, no other genetic testing. | 10 |
| No genetic data | 32, 35, 49, 56, 62, 63, 68, 80,81,82, 85,86,87 |
| No individual case data | 41,43,44,45 |
| Inadequate genetic/clinical correlation | 53, 58, 60 |
| *C9orf72* expansions of unclear significance or incidental and not clearly causal. | 50, 51,52,57, 76, 84 |
| Review | 64, 65 |
| No abstract. No human subjects clinical information | 77 |
