# Supplementary material for: Characterization of Movement Disorder Phenomenology in Genetically Proven, Familial Frontotemporal Lobar Degeneration: A Systematic Review and Meta-Analysis
Source: PLoS One. 2016 Apr 21;11(4):e0153852. doi: 10.1371/journal.pone.0153852 (PMC4839564; doi:10.1371/journal.pone.0153852)
Supplement: S2 Table — (DOCX) [file pone.0153852.s005.docx]

**Supplementary table 2. Number of studies for each outcome.**

|  | **MAPT**  **No. Studies ^A^** | **PGRN**  **No. Studies ^A^** | **C0ORF72**  **No. Studies ^A^** | **OVERALL**  **No. Studies ^A^** |
| --- | --- | --- | --- | --- |
| **Proportion of Males** | 15 | 8 | 8 | 31 |
| **Movement Disorder** | 16 | 10 | 9 | 35 |
| **Non-movement Disorder** | 15 | 10 | 8 | 33 |
| **Movement + Non-movement Disorder** | 15 | 10 | 8 | 33 |
| **Behavioural Disorder** | 15 | 10 | 8 | 33 |
| **Cognitive Disorder** | 15 | 10 | 8 | 33 |
| **Language Disorder** | 15 | 10 | 8 | 33 |
| **Behavioural + Cognitive** | 15 | 10 | 8 | 33 |
| **Behavioural + Language** | 15 | 10 | 8 | 33 |
| **Cognitive + Language** | 15 | 10 | 8 | 33 |
| **PSPS** | 17 | 10 | 9 | 36 |
| **CBS** | 16 | 8 | 10 | 34 |
| **Parkinsonism** | 21 | 10 | 10 | 41 |
| **L-dopa response absent** | 5 | 0 | 2 | 7 |
| **L-dopa response partial** | 6 | 0 | 2 | 7 |
| **L-dopa response present** | 6 | 0 | 2 | 7 |

^A^ Number of studies for each outcome presented in table excludes case studies (studies with a single patient). For the main analysis, the total number of studies used for an outcome was the number presented in the table plus case studies pooled together into a single artificial study with the corresponding genetic mutation. For example, for the overall estimate for movement onset, 35 studies plus the artificial study consisting of case studies, for a total of 36 studies, were used for the meta-analysis.
