# Supplementary material for: Characterization of Movement Disorder Phenomenology in Genetically Proven, Familial Frontotemporal Lobar Degeneration: A Systematic Review and Meta-Analysis
Source: PLoS One. 2016 Apr 21;11(4):e0153852. doi: 10.1371/journal.pone.0153852 (PMC4839564; doi:10.1371/journal.pone.0153852)
Supplement: S3 Table — (DOCX) [file pone.0153852.s006.docx]

**Supplementary table 3. Non-motor presentations**

|  | **MAPT**  **% (95% CI)** | **PGRN**  **% (95% CI)** | **C9ORF72**  **% (95% CI)** | **Overall**  **% (95% CI)** |
| --- | --- | --- | --- | --- |
| **Behavioural Disorder** | 35.6  (17.0-56.6) | 39.1  (21.4-58.4) | 33.0  (17.6-50.6) | 35.7  (24.4-47.9) |
| **Cognitive Disorder** | 17.0  (7.7-29.0) | 14.3  (4.8-27.7) | 9.8  (3.1-19.6) | 14.4  (8.7-21.2) |
| **Language Disorder** | 5.4  (2.2-10.0) | 18.7  (11.5-27.3) | 9.2  (2.8-18.7) | 9.9  (6.6-13.8) |
| **Behavioural + Cognitive** | 4.8  (1.8-9.2) | 9.3  (2.2-20.6) | 9.8  (3.1-19.5) | 7.7  (4.4-11.8) |
| **Behavioural + Language** | 4.3  (1.5-8.5) | 7.7  (3.2-14.0) | 4.7  (0.6-12.4) | 5.4  (3.0-8.5) |
| **Cognitive + Language** | 3.5  (1.0-7.3) | 6.9  (2.6-12.9) | 6.0  (1.2-14.3) | 4.7  (2.5-7.6) |
