# Supplementary material for: Characterization of Movement Disorder Phenomenology in Genetically Proven, Familial Frontotemporal Lobar Degeneration: A Systematic Review and Meta-Analysis
Source: PLoS One. 2016 Apr 21;11(4):e0153852. doi: 10.1371/journal.pone.0153852 (PMC4839564; doi:10.1371/journal.pone.0153852)
Supplement: S4 Table — (DOCX) [file pone.0153852.s007.docx]

**Supplementary table 4: Levodopa responsiveness**

|  | **MAPT**  **% (95% CI)** | **PGRN**  **% (95% CI)** | **C9ORF72**  **% (95% CI)** | **Overall**  **% (95% CI)** |
| --- | --- | --- | --- | --- |
| **L-dopa response absent** | 62.8  (31.1-89.4) | 75.5  (36.9-98.6) ^A^ | 21.5  (2.4-52.4) | 50.9  (23.3-78.3) |
| **L-dopa response partial** | 25.9  (8.6-48.5) | 24.5  (1.4-63.1) ^A^ | 19.8  (0.9-72.7) | 21.9  (7.7-40.8) |
| **L-dopa response present** | 11.1  (3.4-22.4) | 4.4  (3.6-32.8) ^A^ | 34.3  (0.6-85.0) | 15.3  (4.2-31.6) |

^A^ Estimate from a single pooled study made from case studies

Note: 63 studies missing data on L-dopa response necessary to calculate percentage with type of response. 57 studies did not report any information. 7 studies reported incomplete data on L-dopa response. In total there was information on L-dopa responsiveness for 63 patients.
