# Supplementary material for: Characterization of Movement Disorder Phenomenology in Genetically Proven, Familial Frontotemporal Lobar Degeneration: A Systematic Review and Meta-Analysis
Source: PLoS One. 2016 Apr 21;11(4):e0153852. doi: 10.1371/journal.pone.0153852 (PMC4839564; doi:10.1371/journal.pone.0153852)
Supplement: S5 Table — (DOCX) [file pone.0153852.s008.docx]

**Supplementary table 5. Quality of studies ^A^**

|  | **MAPT**  **Total N=40** | **PGRN**  **Total N=25** | **C9ORF72 Total N=16** | **Overall**  **Total N=81** |
| --- | --- | --- | --- | --- |
|  | **N (%)** | **N (%)** | **N (%)** | **N (%)** |
| **Sample size N≥5** | 11 (27.5%) | 5 (20.0%) | 5 (31.3%) | 21 (25.9%) |
| **Movement disorder details of first 3 years** | 28 (70.0%) | 17 (68.0%) | 11 (68.8%) | 56 (69.1%) |
| **Follow up ≥ 5 years** | 17 (42.5%) | 14 (56.0%) | 5 (31.3%) | 36 (44.4%) |

^A^ Artificial study consisting of pooled case studies (reports on single patients) not used. Cast studies were assessed individual along with case series (studies with two or more patients).
