# Supplementary material for: Characterization of Movement Disorder Phenomenology in Genetically Proven, Familial Frontotemporal Lobar Degeneration: A Systematic Review and Meta-Analysis
Source: PLoS One. 2016 Apr 21;11(4):e0153852. doi: 10.1371/journal.pone.0153852 (PMC4839564; doi:10.1371/journal.pone.0153852)
Supplement: S6 Table — (DOCX) [file pone.0153852.s009.docx]

**Supplementary table 6. Quantifying heterogeneity of pooled estimates.**

|  | **I^2^ (95% CI)** | **H (95% CI)** |
| --- | --- | --- |
| **Proportion of Males** | 24.5 (0.0-51.4) | 1.1 (0.9-1.4) |
| **Movement Disorder** | 73.8 (63.7-81.1) | 2.0 (1.7-2.3) |
| **Non-movement Disorder** | 75.4 (65.8-82.3) | 2.0 (1.7-2.4) |
| **Movement + Non-movement Disorder** | 0.0 (0.0-38.7) | 1.0 (0.8-1.3) |
| **Behavioural Disorder** | 73.2 (62.4-80.9) | 1.9 (1.6-2.3) |
| **Cognitive Disorder** | 47.4 (21.4-64.8) | 1.4 (1.1-1.7) |
| **Language Disorder** | 0.0 (0.0-38.7) | 1.0 (0.8-1.3) |
| **Behavioural + Cognitive** | 16.9 (0.0-45.8) | 1.1 (0.9-1.4) |
| **Behavioural + Language** | 0.0 (0.0-38.7) | 1.0 (0.8-1.3) |
| **Cognitive + Language** | 0.0 (0.0-38.7) | 1.0 (0.8-1.3) |
| **PSPS** | 69.0 (56.5-77.9) | 1.8 (1.5-2.1) |
| **CBS** | 25.6 (0.0-51.2) | 1.2 (0.9-1.4) |
| **Parkinsonism** | 80.6 (74.4-85.3) | 2.3 (2.0-2.6) |
| **L-dopa response absent** | 78.2 (57.0-88.9) | 2.1 (1.5-3.0) |
| **L-dopa response partial** | 58.3 (8.8-81.0) | 1.5 (1.0-2.3) |
| **L-dopa response present** | 49.4 (0.0-77.4) | 1.4 (0.9-2.1) |
