# Supplementary material for: Characterization of Movement Disorder Phenomenology in Genetically Proven, Familial Frontotemporal Lobar Degeneration: A Systematic Review and Meta-Analysis
Source: PLoS One. 2016 Apr 21;11(4):e0153852. doi: 10.1371/journal.pone.0153852 (PMC4839564; doi:10.1371/journal.pone.0153852)
Supplement: S7 Table — (DOCX) [file pone.0153852.s010.docx]

**Supplementary table 7. Examining impact of substantial heterogeneity on pooled estimates.**

|  | **Original**  **Overall % (95% CI)** | **Heterogeneity Analysis**  **Overall % (95% CI) ^A^** | **% difference** |
| --- | --- | --- | --- |
| **Proportion of Males** | 45.7 (37.9-53.7) | 47.2 (26.9-68.1) | 1.5 |
| **Movement Disorder** | 27.1 (17.4-37.9) | 27.6 (13.1-45.1) | 0.5 |
| **Non-movement Disorder** | 66.5 (54.0-78.0) | 66.1 (46.6-83.0) | -0.4 |
| **Movement + Non-movement Disorder** | 7.7 (4.8-11.1) | 8.8 (1.1-22.8) | 1.1 |
| **Behavioural Disorder** | 35.7 (24.4-47.9) | 35.5 (18.2-55.0) | -0.2 |
| **Cognitive Disorder** | 14.4 (8.7-21.2) | 15.9 (4.5-32.5) | 1.5 |
| **Language Disorder** | 9.9 (6.6-13.8) | 10.3 (1.7-25.1) | 0.4 |
| **Behavioural + Cognitive** | 7.7 (4.4-11.8) | 8.7 (1.0-22.7) | 1.0 |
| **Behavioural + Language** | 5.4 (3.0-8.5) | 6.3 (0.3-19.0) | 0.9 |
| **Cognitive + Language** | 4.7 (2.5-7.6) | 6.5 (0.4-19.2) | 1.8 |
| **PSPS** | 12.2 (6.2-19.7) | 12.9 (3.6-26.7) | 0.7 |
| **CBS** | 10.7 (6.7-15.4) | 12.5 (3.1-27.0) | 1.8 |
| **Parkinsonism** | 79.8 (69.7-88.2) | 79.8 (65.8-90.8) | 0.0 |
| **L-dopa response absent** | 50.9 (23.3-78.3) | 50.6 (12.6-88.1) | -0.3 |
| **L-dopa response partial** | 21.9 (7.7-40.8) | 20.9 (0.3-60.5) | -1.0 |
| **L-dopa response present** | 15.3 (4.2-31.6) | 17.2 (0.0-57.5) | 1.9 |

^A^ Variation in pooled estimates due to statistical heterogeneity (I^2^) set to 90%
