# Supplementary material for: Characterization of Movement Disorder Phenomenology in Genetically Proven, Familial Frontotemporal Lobar Degeneration: A Systematic Review and Meta-Analysis
Source: PLoS One. 2016 Apr 21;11(4):e0153852. doi: 10.1371/journal.pone.0153852 (PMC4839564; doi:10.1371/journal.pone.0153852)
Supplement: S8 Table — (DOCX) [file pone.0153852.s011.docx]

**Supplementary table 8. Examining impact of removing pooled case studies.**

|  | **Overall**  **% (95% CI)** | **Heterogeneity**  **I^2^ (95% CI)**  **H (95% CI)** | **Heterogeneity Analysis**  **Overall % (95% CI) ^A^** |
| --- | --- | --- | --- |
| **Behavioural Disorder** | 45.9 (37.4-54.5) | **I^2^:** 26.9 (0.0-53.2)  **H:** 1.2 (0.9-1.5) | 47.4 (25.5-69.8) |
| **Cognitive Disorder** | 26.8 (16.7-38.3) | **I^2^:** 74.0 (63.8-81.3)  **H:** 2.0 (1.7-2.3) | 27.4 (12.3-45.9) |
| **Language Disorder** | 67.1 (54.0-79.1) | **I^2^:** 74.9 (64.9-82.1)  **H:** 2.0 (1.7-2.4) | 66.6 (45.9-84.4) |
| **Behavioural + Cognitive** | 6.7 (3.8-10.3) | **I^2^:** 0.0 (0.0-39.21)  **H:** 1.0 (0.8-1.3) | 8.6 (0.8-23.7) |
| **Behavioural + Language** | 36.0 (23.9-49.0) | **I^2^:** 73.8 (63.1-81.4)  **H:** 2.0 (1.6-2.3) | 35.7 (17.4-56.5) |
| **Cognitive + Language** | 14.5 (8.4-21.9) | **I^2^:** 48.8 (23.3-65.9)  **H:** 1.4 (1.1-1.7) | 16.0 (4.0-33.9) |
| **Behavioural Disorder** | 10.0 (6.4-14.2) | **I^2^:** 0.0 (0.0-39.2)  **H:** 1.0 (0.8-1.3) | 10.4 (1.4-26.3) |
| **Cognitive Disorder** | 8.2 (4.6-12.7) | **I^2^:** 17.8 (0.0-46.7)  **H:** 1.1 (0.9-1.4) | 8.9 (0.9-24.1) |
| **Language Disorder** | 5.2 (2.7-8.5) | **I^2^:** 0.0 (0.0-39.2)  **H:** 1.0 (0.8-1.3) | 6.3 (0.2-20.0) |
| **Behavioural + Cognitive** | 5.7 (3.1-9.0) | **I^2^:** 0.0 (0.0-39.2)  **H:** 1.0 (0.8-1.3) | 6.8 (0.3-20.8) |
| **Behavioural + Language** | 12.3 (6.0-20.5) | **I^2^:** 69.8 (57.6-78.5)  **H:** 1.8 (1.5-2.2) | 13.0 (3.3-27.8) |
| **Cognitive + Language** | 9.2 (5.7-13.5) | **I^2^:** 13.9 (0.0-43.6)  **H:** 1.1 (0.9-1.3) | 12.2 (2.6-27.5) |
| **Behavioural Disorder** | 79.2 (68.6-88.0) | **I^2^:** 80.1 (73.6-85.0)  **H:** 2.2 (1.9-2.6) | 79.3 (64.4-90.9) |
| **Cognitive Disorder** | 48.4 (16.4-81.1) | **I^2^:** 80.8 (61.2-90.5)  **H:** 2.3 (1.6-3.2) | 48.2 (7.7-90.3) |
| **Language Disorder** | 18.8 (4.4-40.1) | **I^2^:** 59.3 (6.3-82.3)  **H:** 1.6 (1.0-2.4) | 18.4 (0.1-63.1) |
| **Behavioural + Cognitive** | 19.4 (6.1-37.8) | **I^2^:** 42.3 (0.0-75.7)  **H:** 1.3 (0.9-2.0) | 20.7 (0.0-67.7) |

^A^ Variation in pooled estimates due to statistical heterogeneity (I^2^) set to 90%
